# Delusional infestation: Two case reports

**DOI:** 10.1192/j.eurpsy.2021.2034

**Published:** 2021-08-13

**Authors:** C. Peixoto, D. Rego, M. Bicho, J. Mendes Coelho, H. Medeiros

**Affiliations:** Psychiatry, Hospital do Divino Espírito Santo de Ponta Delgada, E.P.E., Ponta Delgada, Ponta Delgada, Portugal

**Keywords:** Delusional Infestation, Delusional parasitosis, Ekbom syndrome

## Abstract

**Introduction:**

Delusional infestation (DI), also known as delusional parasitosis or Ekbom syndrome, is a rare disorder, characterised by fixed belief that the skin, body or immediate environment is infested by small pathogens, despite the lack of any medical evidence for it.

**Objectives:**

To describe and discuss two clinical cases of DI, in order to show two different ways of presenting in this entity.

**Methods:**

Two case report and non-systematic review.

**Results:**

We present the case of a 76-year-old woman, without psychiatric history, with an DI with 5 years of evolution, referred to a psychiatric consultation by a dermatologist. The second case, is a 41-year-old woman with a history of multiple substance use disorder, with an DI with a month of evolution, who resorted to the emergency department. DI is not a single diagnostic entity. The classic form, as represented in the first case,is a primary form, which develops without any known cause or underlying disease, corresponding to a persistent delusional disorder. However, about 60% of patients have secondary forms of DI, in the context of substance misuse, some medications or in the course of physical or psychiatric diseases (e.g. stroke, delirium, dementia, depression, schizophrenia).

**Conclusions:**

DI can occur as a primary delusional disorder or secondary to several other medical conditions. An in-depth clinical history is essential in order to make the correct diagnosis. A multidisciplinary approach is also important, to exclude any possible organic etiology, not forgetting that many patients may turn to other medical specialities first.

**Disclosure:**

No significant relationships.

